# Examining differential item functioning and group differences across student and community samples on the Personality Inventory for the DSM-V-Short Form

**DOI:** 10.4102/ajopa.v7i0.175

**Published:** 2025-09-15

**Authors:** Casper J.J. van Zyl

**Affiliations:** 1 Department of Psychology, Faculty of Humanities, University of Johannesburg, Johannesburg, South Africa

**Keywords:** differential item response theory, group differences, PID-5, community, students

## Abstract

This study forms one part of a larger project of evaluating the Personality Inventory for the Diagnostic and Statistical Manual-V (DSM-V) Short Form (PID-5-SF) in the South African context. Among others, its objectives include investigating its psychometric properties and the development of preliminary norms. For the project, non-clinical adult data were collected from a community sample (*n* = 729) and from students (*n* = 629). To determine if the data can be combined into a single adult sample for further research, this initial study seeks to investigate if there are any differences across the student and community samples. No meaningful group differences would suggest that the data can be merged, while differences would suggest that the student and community data should be further analysed separately. To that end, the present study investigated: (1) Differential Item Functioning (DIF) and (2) group differences on the PID-5-SF across the student and community data. Analyses using the ‘lordif’ package in R found no meaningful DIF based on McFadden’s pseudo-R-square thresholds. In addition, no substantive group differences were observed on any of PID-5-SF’s 25 facets.

**Contribution:**

This study shows that student data collected on the PID-5-SF can, in the present case, be considered representative of the broader community. In turn, this facilitates further ongoing work on its psychometric properties and the development of preliminary norms. In this way, it will contribute to the international literature on the PID-5-SF’s psychometric functioning and enable further applied research on personality disorders among practitioners and researchers in South Africa.

## Introduction

The diagnosis of personality disorders has been an ongoing challenge in clinical psychology and psychiatry for a long time (Frances, 1982, 1993; Walton & Presley, 1973; Widiger & Trull, 2007; Zachar et al., 2016). A major reason for this is that personality disorders are typically classified using categorical models as specified in various versions of established classification systems such as the Diagnostic and Statistical Manual of Mental Disorders (DSM). However, the issues surrounding the use of such categorical approaches are numerous. Some of the major issues include, for example, the extreme comorbidity among disorders, within-disorder heterogeneity, the use of arbitrary diagnostic thresholds, unsatisfactory reliability and validity and the overuse of the Personality Disorder Not Otherwise Specified diagnosis (Huprich, 2015; Skodol, 2012; Trull & Durrett, 2005; Widiger & Trull, 2007).

While it was long recognised that this approach is problematic and required change, the process to develop and implement a new diagnostic system to replace the existing one was complex (Clark, 2007; Zacher et al., 2016). Because of the challenges encountered during the development of the dimensional model that was to replace the existing system, the categorical approach was retained in the fifth version of the DSM (DSM-V). Instead of replacing the old, the new model was presented as the ‘Alternative DSM-V model for Personality Disorders (AMPD)’ under the heading ‘Emerging Measures and Models’ in Section III of the DSM-V. For the time being then, DSM-V allows continuity with current practice, by enabling clinicians to use either the old classification system or they can choose to use the new dimensional model (Freilich et al., 2023).

The AMPD comprises two main criteria – A and B. Criterion A assesses self and interpersonal functioning, and criterion B contains a measure of maladaptive personality traits – the Personality Inventory for the Diagnostic and Statistical Manual of Mental Disorders (PID-5). The PID-5 evaluates individuals on five broad trait domains: Negative Affectivity, Detachment, Antagonism, Disinhibition and Psychoticism. These domains are further broken down into 25 lower-order trait facets, providing a comprehensive and nuanced assessment of personality functioning. Thus, the AMPD emphasises the assessment of personality traits on a continuum, recognising that maladaptive personality traits exist along a spectrum and may be present to varying degrees across individuals. This approach is also conceptually consistent with well-established and replicated models of normal-range personality (Maples et al., 2015).

According to Dankaert (2024), the introduction of the AMPD and the PID-5, in particular, represents a significant shift in the understanding and assessment of personality disorders. By focusing on traits rather than categories, the inventory allows clinicians and researchers to capture the full range of personality pathology, from subtle maladaptive tendencies to severe personality dysfunction. This trait-based approach not only offers a more flexible framework for diagnosis and treatment but also provides a foundation for understanding how personality traits interact and contribute to overall psychological functioning.

While many studies have found the PID-5 to be comprehensive and psychometrically robust (Freilich et al., 2023; Somma et al., 2019; Zimmermann et al., 2019), it is unfortunately quite lengthy – a 220-item measure. This is likely to be prohibitive for everyday use, for instance, as part of a consultation session. While there is also a brief form of the measure (PID-5-BF), this version, by contrast, has been criticised as lacking depth (Anderson et al., 2018), given that it contains only 25 items measuring the domains with no facet-level information.

To address the length constraints of the PID-5 and the limited depth of the PID-5-BF, Maples et al. (2015) developed a shorter version called the Personality Inventory for DSM-5-Short-Form (PID-5-SF). With just 100 items, the PID-5-SF effectively retains the depth and breadth of the original PID-5 without loss of information (Maples et al., 2015). To use the measure appropriately in the South African context, however, it is essential to ensure that it is psychometrically sound and appropriately standardised for this setting. This study contributes to a project aimed at achieving precisely that. In particular, it seeks to investigate the PID-5-SF’s psychometric properties and to develop preliminary norms, which will make it more appropriate for use within the South African setting given that the current generic method of scoring recommended by the APA (American Psychiatric Association [APA], 2023) is not ideal. Towards that end, the present study aims to first determine whether the project’s data, collected from two distinct non-clinical adult samples – one student and one community – can be combined into a single, larger dataset (Dankaert, 2024).

Merging datasets from different populations is a common practice in research to increase sample size, improve statistical power and enhance generalisability of findings. However, to justify merging the two datasets – one from a community sample and one from a student sample – it is essential to first evaluate whether the samples can be treated as comparable. This involves examining observed score differences between the two samples, as significant differences would indicate that the student sample is distinct and merging the data would not be warranted. In contrast, no meaningful differences across the groups will support merging of the data. However, appropriate mean score comparisons require first establishing measurement equivalence across groups.

Differential item functioning (DIF) studies provide a robust framework to detect whether items are biased (deviate systematically) because of factors unrelated to latent construct. The absence of DIF ensures that any differences in scores reflect true differences in the construct being measured rather than artefacts of the measurement process (Berrío et al., 2020; Osterlind & Everson, 2009). Uniform DIF occurs when an item consistently favours one group across all levels of the latent construct, while non-uniform DIF indicates that the item’s performance varies across latent levels between groups. By confirming the absence of meaningful DIF, we can establish measurement equivalence, thereby validating the comparison of mean scores (Tay et al., 2015).

The present study therefore seeks to determine whether the community and student data can be merged. This will be achieved by: (1) investigating uniform and non-uniform DIF across the student and community samples and (2) examining for observed mean score differences across the groups. The outcome of this work will set the stage for subsequent research within the larger project, of which this study represents one part. It will determine whether further analyses, which include investigating the psychometric properties of the PID-5-SF and developing preliminary norms for the assessment in South Africa, should be done on a single adult dataset (by merging the data from the community and student samples) or whether the data should be analysed separately.

## Method

### Participants

The sample contained 1358 participants in total, collected primarily from two sources, a community sample and a student sample. The community sample comprised 729 participants with a mean age of 34.8 years (standard deviation [s.d.] = 10.6 years), of which 404 were women and 325 were men. The sample composition was racially diverse with representation as follows based on self-identification: black people (31.4%), white people (12.6%), Indian people (2.5%), Asian people (0.2%), multi-racial people (0.2%), coloured people = 6.6% and other (0.2%). The student sample contained 629 participants. The mean age was 26.5 years (s.d. = 10.2 years), including 448 women and 181 men. Demographic representation included the following: black people (28.4%), white people (14%), Indian people (1.2%), Asian people (0.1%), multi-racial people = (0.4%), coloured people = 2% and other (0.1%).

### Measure

The PID-5 Short Form consists of 100 items that evaluate five broad domains. Each item is rated on a 4-point Likert scale ranging from 0 (very false or often false) to 3 (very true or often true), reflecting the extent to which each statement describes the respondent’s thoughts, feelings and behaviours. The five broad domains measured by the PID-5 correspond to personality traits associated with dysfunctions in self and interpersonal functioning, as proposed in the DSM-5 model. The measure comprises 25 narrower facets, grouped into the five broad domains (Maples et al., 2015) as follows: *Negative Affectivity* (emotional liability, anxiousness, separation insecurity, submissiveness, hostility, perseveration); *Detachment* (withdrawal, intimacy avoidance, anhedonia, depressivity, restricted affectivity, suspiciousness); *Antagonism* (manipulativeness, deceitfulness, grandiosity, attention seeking, callousness); *Disinhibition* (irresponsibility, impulsivity, distractibility, risk taking, rigid perfectionism (reversed)); *Psychoticism* (unusual beliefs and experiences, eccentricity, cognitive and perceptual dysregulation).

### Procedure

The data were collected as part of PhD thesis (Dankaert, 2024). All adults (18 years and older) were eligible to participate in the study. The student sample included 629 psychology students. The questionnaire was broadly distributed among psychology students, with third-year and fourth-year students earning course credit for participation while others took part voluntarily. The community sample consisted of 729 adults recruited *via* a survey company, where participants completed the questionnaire for compensation based on the eligibility criteria set by the researcher. None of the questionnaires were completed under supervision. Informed consent was obtained from all the participants.

### Data analysis

Revelle and Condon’s (2025) ‘unidim’ (*u*) index was computed in *R* to assess each of the 25 facets for unidimensionality for the student and community samples, respectively. This index has shown excellent sensitivity to general factor saturation and less sensitivity to the number of test items, which makes it ideal for short four-item scales where other good options like omega hierarchical do not perform as well. The ‘lordif’ package (Choi et al., 2011) in *R* (R Core Team, 2024) was used to examine for DIF. The ‘lordif’ package conducts DIF analysis through an iterative process that merges Ordinal Logistic Regression (OLR) with Item Response Theory (IRT). It employs nested OLR models where each item is evaluated for DIF by comparing a base model, which includes only main effects, against a nested model that adds interaction terms between item responses and group membership to detect both uniform and non-uniform DIF. Chi-square tests of statistical significance and pseudo *R*-square values are reported to evaluate the presence and magnitude of DIF (Choi et al., 2011). While DIF is always present to some extent, what is important is the size and direction of DIF. This study sought to examine the magnitude of DIF across all items, expecting it to be small enough to be negligible across the groups. It further aimed to ensure that several small effects in the same direction are not having a cumulative effect at scale level. Differential item functioning was considered for each facet separately. Group differences were then evaluated in R and the ‘effect size’ (Ben-Shachar et al., 2020) package was used to produce Cohen’s *d* effect size estimates.

### Ethical considerations

The study was conducted in accordance with the guidelines of the Declaration of Helsinki. The study obtained ethical clearance from the Humanities Ethics Committee of the University of Johannesburg (Reference Number: REC-01-090-2020).

## Results

Table 1 shows *u*-values ranging from 0.88 and higher indicating strong support for the unidimensionality of the 25 facets, estimated separately for the student and community samples by facet. The default setting of the ‘lordif’ package for detecting DIF is an alpha level of 0.01 for statistical significance. However, a well-known issue in frequentist statistics is that small and insignificant differences will be flagged for statistical significance when samples are large (Greenland et al., 2016). To mitigate this problem, ‘lordif’ also includes several pseudo *R*-square indicators of effect size such as McFadden’s, Nagelkerke and CoxSnell. The corresponding effect sizes for McFadden’s pseudo *R*^2^ are: < 0.13 ‘negligible’, 0.13–0.26 ‘moderate’ and > 0.26 ‘large’ (Zumbo, 1999). For the purpose of this study, McFadden’s pseudo *R*^2^ values ≥ 0.13 was used as the threshold for meaningful DIF that required further investigation. However, it is possible that despite not meeting this threshold for DIF, the unique nature of smaller amounts of DIF across one or more items can have a meaningful effect at scale level. For this reason, effects at scale level will be considered regardless of whether the DIF threshold for any one item was violated. The ‘lordif’ package facilitates this by generating a Test Characteristic Curve plot that allows comparison of the scale level effect with and without accommodating for DIF.

**TABLE 1:** Chi-square and pseudo *R*-square values for tests of uniform and non-uniform differential item functioning.

Item	*χ*^2^ 12	*χ*^2^ 13	*χ*^2^ 23	McFadden’s pseudo *R*^2^ 12	McFadden’s pseudo *R*^2^ 13	McFadden’s pseudo *R*^2^ 23
**Anhedonia (*u*_s_ = 0.99, *u*_c_ = 0.98)**						
1	0.0101	0.0018	0.0143	0.0020	0.0038	0.0018
2	0.1129	0.0589	0.0759	0.0008	0.0018	0.0010
3	0.1026	0.2147	0.5213	0.0008	0.0009	0.0001
4	0.0000	0.0000	0.0023	0.0063	0.0093	0.0030
**Anxiousness (*u*_s_ = 0.99, *u*_c_ = 0.99)**						
1	0.5512	0.6410	0.4648	0.0001	0.0002	0.0001
2	0.4324	0.7272	0.8859	0.0002	0.0002	0.0000
3	0.0365	0.0196	0.0619	0.0012	0.0021	0.0009
4	0.0233	0.0498	0.3545	0.0014	0.0016	0.0002
**Attention seeking (*u*_s_ = 0.99, *u*_c_ = 1.00)**						
1	0.9685	0.7709	0.4713	0.0000	0.0002	0.0002
2	0.7788	0.5725	0.3086	0.0000	0.0003	0.0003
3	0.6531	0.6218	0.3870	0.0001	0.0003	0.0002
4	0.0000	0.0000	0.6578	0.0132	0.0133	0.0001
**Callousness (*u*_s_ = 0.99, *u*_c_ = 0.97)**						
1	0.9900	0.2578	0.0997	0.0000	0.0014	0.0014
2	0.3811	0.6642	0.8213	0.0004	0.0004	0.0000
3	0.0858	0.2216	0.8040	0.0016	0.0016	0.0000
4	0.8754	0.4262	0.1948	0.0000	0.0007	0.0007
**Deceitfulness (*u*_s_ = 0.98, *u*_c_ = 0.98)**						
1	0.8568	0.8949	0.6633	0.0000	0.0001	0.0001
2	0.4987	0.3767	0.2215	0.0002	0.0007	0.0006
3	0.0413	0.1224	0.8456	0.0018	0.0018	0.0000
4	0.0679	0.0436	0.0869	0.0010	0.0018	0.0009
**Depressivity (*u*_s_ = 0.99, *u*_c_ = 0.98)**						
1	0.0025	0.0050	0.2256	0.0039	0.0046	0.0006
2	0.3035	0.4756	0.5132	0.0003	0.0004	0.0001
3	0.8804	0.2509	0.0977	0.0000	0.0010	0.0010
4	0.0000	0.0001	0.3107	0.0082	0.0086	0.0005
**Distractibility (*u*_s_ = 1.00, *u*_c_ = 1.00)**						
1	0.3826	0.0013	0.0004	0.0002	0.0037	0.0034
2	0.0000	0.0000	0.0352	0.0111	0.0123	0.0012
3	0.2252	0.4152	0.5923	0.0004	0.0005	0.0001
4	0.4032	0.5011	0.4085	0.0002	0.0004	0.0002
**Eccentricity (*u*_s_ = 0.99, *u*_c_ = 1.00)**						
1	0.9666	0.8485	0.5675	0.0000	0.0001	0.0001
2	0.9072	0.9345	0.7271	0.0000	0.0000	0.0000
3	0.1432	0.1331	0.1691	0.0006	0.0011	0.0005
4	0.2592	0.1400	0.1029	0.0003	0.0011	0.0007
**Emotional liability (*u*_s_ = 0.96, *u*_c_ = 0.97)**						
1	0.0527	0.1459	0.7577	0.0010	0.0011	0.0000
2	0.1003	0.2166	0.5491	0.0008	0.0009	0.0001
3	0.7701	0.9553	0.9377	0.0000	0.0000	0.0000
4	0.8773	0.7057	0.4119	0.0000	0.0002	0.0002
**Grandiosity (*u*_s_ = 0.98, *u*_c_ = 0.96)**						
1	0.6334	0.8102	0.6599	0.0001	0.0001	0.0001
2	0.1417	0.3357	0.8771	0.0008	0.0008	0.0000
3	0.4986	0.0017	0.0004	0.0001	0.0036	0.0035
4	0.0000	0.0000	0.0095	0.0075	0.0098	0.0022
**Hostility (*u*_s_ = 0.90, *u*_c_ = 0.88)**						
1	0.5454	0.2968	0.1508	0.0001	0.0007	0.0006
2	0.9174	0.5168	0.2525	0.0000	0.0004	0.0004
3	0.6880	0.7510	0.5213	0.0000	0.0002	0.0001
4	0.0000	0.0000	0.8218	0.0074	0.0074	0.0000
**Impulsivity (*u*_s_ = 0.99, *u*_c_ = 0.99)**						
1	0.6412	0.7295	0.5201	0.0001	0.0002	0.0001
2	0.2024	0.3588	0.5144	0.0005	0.0007	0.0001
3	0.3144	0.5852	0.8073	0.0003	0.0003	0.0000
4	0.7661	0.9566	0.9897	0.0000	0.0000	0.0000
**Intimacy avoidance (*u*_s_ = 0.98, *u*_c_ = 0.99)**						
**1**	0.5321	0.4143	0.2415	0.0001	0.0005	0.0004
**2**	0.0000	0.0000	0.0020	0.0073	0.0101	0.0028
**3**	0.9657	0.9557	0.7656	0.0000	0.0000	0.0000
**4**	0.0067	0.0002	0.0022	0.0022	0.0050	0.0028
**Irresponsibility (*u*_s_ = 0.95, *u*_c_ = 0.99)**						
**1**	0.1684	0.3418	0.6170	0.0007	0.0008	0.0001
**2**	0.6439	0.0000	0.0000	0.0001	0.0143	0.0142
**3**	0.0000	0.0000	0.0000	0.0080	0.0158	0.0079
**4**	0.8956	0.9381	0.7394	0.0000	0.0001	0.0001
**Manipulativeness (*u*_s_ = 0.97, *u*_c_ = 0.92)**						
**1**	0.1131	0.0183	0.0191	0.0007	0.0023	0.0016
**2**	0.6581	0.3375	0.1598	0.0001	0.0006	0.0006
**3**	0.5286	0.5726	0.3968	0.0002	0.0005	0.0003
**4**	0.1399	0.1100	0.1349	0.0009	0.0017	0.0009
**Perceptual dysregulation (*u*_s_ = 0.96, *u*_c_ = 0.97)**						
**1**	0.0157	0.0139	0.0999	0.0018	0.0027	0.0008
**2**	0.0000	0.0000	0.0204	0.0103	0.0120	0.0017
**3**	0.5602	0.6122	0.4230	0.0001	0.0004	0.0003
**4**	0.0000	0.0000	0.0488	0.0105	0.0116	0.0012
**Perseveration (*u*_s_ = 0.91, *u*_c_ = 0.96)**						
**1**	0.3207	0.4493	0.4333	0.0003	0.0005	0.0002
**2**	0.3848	0.5656	0.5353	0.0002	0.0003	0.0001
**3**	0.1061	0.1546	0.2894	0.0007	0.0010	0.0003
**4**	0.6248	0.8158	0.6820	0.0001	0.0001	0.0001
**Restricted affectivity (*u*_s_ = 0.92, *u*_c_ = 0.91)**						
1	0.0000	0.0000	0.0438	0.0060	0.0073	0.0013
2	0.9907	0.9705	0.8068	0.0000	0.0000	0.0000
3	0.0002	0.0004	0.1826	0.0039	0.0044	0.0005
4	0.2472	0.1720	0.1396	0.0004	0.0010	0.0006
**Rigid perfectionism (*u*_s_ = 0.98, *u*_c_ = 0.98)**						
1	0.9806	0.4845	0.2287	0.0000	0.0004	0.0004
2	0.2312	0.4731	0.8011	0.0004	0.0004	0.0000
3	0.6792	0.7918	0.5865	0.0000	0.0001	0.0001
4	0.2077	0.3739	0.5376	0.0004	0.0006	0.0001
**Risk taking (*u*_s_ = 0.97, *u*_c_ = 0.94)**						
1	0.3009	0.2199	0.1616	0.0004	0.0011	0.0007
2	0.7518	0.8121	0.5738	0.0000	0.0001	0.0001
3	0.9710	0.9009	0.6488	0.0000	0.0001	0.0001
4	0.9455	0.9616	0.7862	0.0000	0.0000	0.0000
**Separation insecurity (*u*_s_ = 0.97, *u*_c_ = 0.93)**						
1	0.2445	0.4435	0.6023	0.0004	0.0004	0.0001
2	0.0000	0.0000	0.1143	0.0054	0.0061	0.0007
3	0.1882	0.0569	0.0454	0.0006	0.0018	0.0013
4	0.7053	0.5831	0.3333	0.0000	0.0003	0.0003
**Submissiveness (*u*_s_ = 0.99, *u*_c_ = 0.99)**						
1	0.9806	0.4845	0.2287	0.0000	0.0004	0.0004
2	0.2312	0.4731	0.8011	0.0004	0.0004	0.0000
3	0.6792	0.7918	0.5865	0.0000	0.0001	0.0001
4	0.2077	0.3739	0.5376	0.0004	0.0006	0.0001
**Suspiciousness (*u*_s_ = 0.97, *u*_c_ = 0.96)**						
1	0.9806	0.4845	0.2287	0.0000	0.0004	0.0004
2	0.2312	0.4731	0.8011	0.0004	0.0004	0.0000
3	0.6792	0.7918	0.5865	0.0000	0.0001	0.0001
4	0.2077	0.3739	0.5376	0.0004	0.0006	0.0001
**Unusual beliefs and experiences (*u*_s_ = 0.93, *u*_c_ = 0.90)**						
1	0.8055	0.9696	0.9724	0.0000	0.0000	0.0000
2	0.5640	0.8165	0.7874	0.0001	0.0001	0.0000
3	0.2423	0.2934	0.2976	0.0004	0.0007	0.0003
4	0.1136	0.2262	0.4935	0.0007	0.0008	0.0001
**Withdrawal (*u*_s_ = 0.99, *u*_c_ = 0.99)**						
1	0.0051	0.0121	0.3159	0.0022	0.0025	0.0003
2	0.9785	0.1389	0.0470	0.0000	0.0012	0.0012
3	0.0000	0.0000	0.2144	0.0066	0.0071	0.0005
4	0.8460	0.9703	0.8805	0.0000	0.0000	0.0000

Note: *u*_s_, unidim coefficient for the student sample; *u*_c_, unidim coefficient for the community sample.

Table 1 shows that several items were flagged for DIF based upon the Chi-square test for statistical significance at the 0.01 level. However, when inspecting effect sizes for these items, the McFadden values were all much smaller than 0.13 and therefore considered negligible.

**FIGURE 1:**
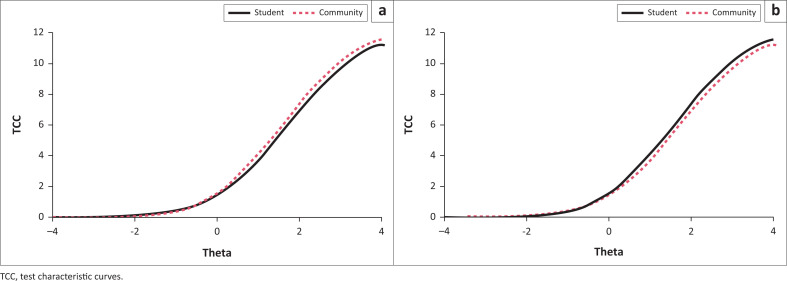
Differential item functioning impact on the test characteristic curves: (a) Irresponsibility; (b) Perceptual dysregulation.

Therefore, no items were flagged for either uniform or non-uniform DIF. While inspecting the test characteristic curves (TCC), the Irresponsibility and Perceptual Dysregulation facets appeared to have small but systematic differences for the expected total scores across the student and community groups at each level of theta. The TCCs for these two facets are presented in Figure 1. Although none of the items on these facets were flagged for meaningful DIF, the small but systematic scale level DIF observed in Figure 1 was nonetheless accounted for by computing calibrated, group-specific person parameters, which were used in subsequent analyses.

### Group differences on the PID-5-SF facets

Group differences were explored between the student and community samples on the 25 facets and 5 domains of the PID-5-SF. The results are presented in Table 2. A Cohen’s *d* value of 0.50 or larger was set as the threshold for a meaningful effect size. This is based on Cohen’s suggested interpretation for a medium effect size (Cohen, 1988). An effect size of this magnitude has a probability of superiority of 63.8%, which means that a random person picked from the higher mean group will have a 63.8% probability of having a higher score than a random person picked from the lower mean group (Magnusson, 2023). Using this criterion, none of the facets or domains appear to have noteworthy mean score differences across the student and community samples. Only small effects were observed for some facets, including Anxiousness, Callousness, Distractibility, Grandiosity, Irresponsibility, Perceptual Dysregulation, Suspiciousness, Unusual Beliefs and Experiences, as well as the Antagonism domain.

**TABLE 2:** Group differences between the student and community samples.

Facet	Student (Mean)	Community (Mean)	Mean difference	*t*-statistic	*p*-value	Cohen’s *d*
Anhedonia	0.876	0.917	−0.04136	−1.0489	0.294	−0.057
Anxiousness	1.73	1.56	0.16692	3.8093	< 0.001	0.207
Attention seeking	0.921	0.907	0.01310	0.3294	0.742	0.018
Callousness	0.280	0.441	−0.16115	−5.7513	< 0.001	−0.313
Deceitfulness	0.607	0.729	−0.12256	−3.5097	< 0.001	−0.191
Depressivity	0.523	0.653	−0.12995	−3.4739	< 0.001	−0.189
Distractibility	1.31	1.06	0.25144	5.7855	< 0.001	−0.167
Eccentricity	1.39	1.43	−0.03553	−0.7967	0.426	−0.043
Emotional liability	1.18	1.09	0.08514	1.9233	0.055	0.105
Grandiosity	0.628	0.855	−0.22770	−6.5368	< 0.001	−0.356
Hostility	0.928	1.00	−0.07474	−1.8641	0.063	−0.101
Impulsivity	0.911	1.01	−0.09961	−2.7466	0.006	−0.149
Intimacy avoidance	0.858	0.938	−0.07982	−1.9057	0.057	−0.104
Irresponsibility	0.455	0.577	−0.12133	−4.0141	< 0.001	−0.143
Manipulativeness	0.760	0.849	−0.08923	−2.5828	0.010	−0.141
Perceptual dysregulation	0.656	0.820	−0.16450	−4.3924	< 0.001	−0.323
Perseveration	1.09	1.06	0.03397	0.8997	0.368	0.049
Restricted affectivity	0.990	1.09	−0.10253	−2.6798	0.007	−0.146
Rigid perfectionism	1.38	1.42	−0.04657	−1.1599	0.246	−0.063
Risk taking	0.794	1.04	0.24880	−6.8447	< 0.001	−0.372
Separation insecurity	1.09	1.09	−0.00430	−0.0995	0.921	−0.005
Submissiveness	0.855	0.809	0.04634	1.2698	0.204	0.069
Suspiciousness	0.984	1.21	−0.22572	−6.4993	< 0.001	−0.354
Unusual beliefs	0.935	1.11	−0.17424	−4.2646	< 0.001	−0.232
Withdrawal	1.10	1.19	−0.08327	−2.0388	0.042	−0.111
Negative affect	1.33	1.25	0.08259	2.3115	0.021	0.126
Detachment	0.946	1.01	−0.06815	−2.0550	0.040	−0.112
Antagonism	0.665	0.811	−0.14650	−4.9903	< 0.001	−0.272
Disinhibition	0.892	0.881	0.01017	0.3393	0.734	0.018
Psychoticism	0.995	1.12	−0.12476	−3.5196	< 0.001	−0.192

## Discussion

The aim of this study was to examine whether the adult data from two samples – student and community – can be combined. Since students might be considered a unique population, it was important to ensure that there were no problematic group differences in this data that would prevent it from being combined with the rest of the community data. To compare the groups meaningfully, it was necessary to confirm that the scores of both groups were not adversely affected by either uniform or non-uniform DIF on any of the 25 facets of the PID-5-SF.

Given that the threshold for DIF was set to a McFadden’s pseudo *R*-square value ≥ 0.13 – constituting non-negligible effects – none of the 100 items from the 25 facets were flagged for either uniform or non-uniform DIF. Despite the fact that no item-level DIF was found, the potential for scale level DIF was still considered separately for each facet. Small but systemic DIF was found for the Perceptual Dysregulation and Irresponsibility facets, although inspection of the TCCs shows that it is probably negligible. However, to err on the side of caution, person parameter scores accounting for this DIF were computed. This ensured that the DIF, albeit marginal, played no role in the subsequent analysis of group differences for these two facets.

Group differences between the student and community samples were investigated across all facets and domains. Several small effects were identified. Students had slightly higher scores on the Anxiousness and Distractibility facets, whereas scores were marginally higher for the community sample on the Callousness, Grandiosity, Irresponsibility, Perceptual Dysregulation, Suspiciousness, Unusual Belief and Experiences facets and the Antagonism domain. However, none came close to the threshold set for substantive interest. Thus, there were no meaningful differences across the student and community samples on any of the facets or domains of the PID-5-SF.

This study shares the view that students cannot simply be assumed to be representative of the broader community (Hanel & Vione, 2016). It therefore sought to empirically examine to what degree the student data collected on the PID-5-SF can be considered representative (or not) of the community data. The investigation of DIF found no evidence for meaningful DIF on any of its 25 facets. There were also no meaningful mean score differences at either the facet or domain levels of the assessment.

The results of this study will enable further research on the PID-5-SF in South Africa. The fact that the data from these two samples can be combined, allows for a larger dataset that can be used in future to further investigate the psychological functioning of the measure.

### Limitations and recommendations for future research

One limitation of this study is that the combined data do not perfectly match the South African population; however, it is nonetheless very diverse and representative with regards to age, gender and race. The data will facilitate further measurement equivalence studies across other groups of interest, where it might be important to examine for mean score differences. For example, in order to develop preliminary norms for the PID-5-SF, it would be necessary to first establish if there are noteworthy differences across ethnicity, age, gender and other subgroups of interest. This larger dataset will also allow for more robust work on its psychometric properties and other substantive questions of clinical interest. In addition to research on the PID-5-SF’s psychometric properties, measurement equivalence, group differences and standardisation, future work will include conducting research on clinical samples to determine if the measure functions well on this population, and to determine if separate norms might be needed. Future studies will also be required on the full and brief versions of the PID-5, since it would be optimal to have a body of research along with norms for each of its versions, so that practitioners have reliable and valid options to choose from, depending on their requirements.

## Conclusion

Combined, the DIF analyses and examination of group differences across the community and student samples support the conclusion that student data can, in this case, be considered representative of the larger community data and that combining the data is therefore justified.
